# Marine Invertebrate Antimicrobial Peptides and Their Potential as Novel Peptide Antibiotics

**DOI:** 10.3390/md21100503

**Published:** 2023-09-23

**Authors:** Svetlana V. Guryanova, Sergey V. Balandin, Oksana Yu. Belogurova-Ovchinnikova, Tatiana V. Ovchinnikova

**Affiliations:** 1 M.M. Shemyakin and Yu.A. Ovchinnikov Institute of Bioorganic Chemistry, Russian Academy of Sciences, 117997 Moscow, Russia; svgur@ibch.ru (S.V.G.); arenicin@mail.ru (S.V.B.); 2 Medical Institute, Peoples’ Friendship University of Russia, 117198 Moscow, Russia; 3 Phystech School of Biological and Medical Physics, Moscow Institute of Physics and Technology, 141701 Dolgoprudny, Russia; belogurova-ovchinnikova.oyu@mipt.ru; 4 Department of Biotechnology, I.M. Sechenov First Moscow State Medical University, 119991 Moscow, Russia

**Keywords:** marine invertebrates, antimicrobial peptides, host defense peptides, innate immunity

## Abstract

Marine invertebrates constantly interact with a wide range of microorganisms in their aquatic environment and possess an effective defense system that has enabled their existence for millions of years. Their lack of acquired immunity sets marine invertebrates apart from other marine animals. Invertebrates could rely on their innate immunity, providing the first line of defense, survival, and thriving. The innate immune system of marine invertebrates includes various biologically active compounds, and specifically, antimicrobial peptides. Nowadays, there is a revive of interest in these peptides due to the urgent need to discover novel drugs against antibiotic-resistant bacterial strains, a pressing global concern in modern healthcare. Modern technologies offer extensive possibilities for the development of innovative drugs based on these compounds, which can act against bacteria, fungi, protozoa, and viruses. This review focuses on structural peculiarities, biological functions, gene expression, biosynthesis, mechanisms of antimicrobial action, regulatory activities, and prospects for the therapeutic use of antimicrobial peptides derived from marine invertebrates.

## 1. Introduction

Marine invertebrates represent a vast variety of animals inhabiting salt water environments. This diverse group encompasses various aquatic organisms, including sponges, corals, cnidarians, polychaeta, mollusks, crustaceans, and echinoderms. A notable feature of marine invertebrates is their remarkable defense mechanisms, developed during long-term evolution in constant contact with pathogenic microorganisms. For instance, one milliliter of sea water contains approximately one million bacteria, ten million viruses, and around one thousand small protozoans and algae, also known as protists [1]. Over several hundred million years, marine invertebrates have evolved robust defense mechanisms to thrive in challenging environments [2]. Remarkably, microorganisms, including pathogens, are more abundant in sea water compared to terrestrial environments [3]. In aggregate, oceanic microorganisms harbor an extensive and untapped reserve of biological and genetic diversity, surpassing any other known on Earth [1,4]. It is noteworthy that marine organisms surpass terrestrial counterparts in terms of both biodiversity and biomass [5]. As a result, there is a prevailing belief that biologically active compounds found in marine organisms far exceed those derived from terrestrial organisms [6].

Unlike vertebrates, marine invertebrates lack acquired immunity but possess effective defensive strategies, which encompass the presence of integuments, such as shells, cuticles, mucus, encapsulation, as well as cellular and humoral factors of innate immunity [7]. Cellular factors involve mobile phagocytic cells, such as hemocytes [8,9]. Innate immunity receptors presenting on the surface of these cells recognize pathogen-associated molecular patterns (PAMPs), being common to diverse microorganism families. This recognition triggers signaling pathways, leading to phagocytosis and the production of antimicrobial substances [10]. Humoral factors encompass antimicrobial proteins and peptides dissolved in coelomic fluid or hemolymph [11,12,13,14].

Antimicrobial peptides (AMPs) have gained the attention of researchers as natural compounds possessing biological activities and significant potential for medical applications. AMPs are crucial components of the innate immune system of marine organisms [15], and now, they are considered not only as effectors of the innate immunity, but also as regulators of proliferative activities and protective processes [16]. For this reason, the more specified term “host defense peptides” is now commonly used along with “antimicrobial peptides”. Initially, AMPs have implied evolutionarily conserved, gene-encoded short peptides comprising 12–45 amino acid residues and typically exhibiting a cationic nature [12,15,16,17]. As the concept of AMPs has since expanded, the current definition is primarily based on physicochemical criteria (length less than 100 amino acids, amphipathic, cationic) and their ability to kill microbes [18]. These amphipathic peptides serve to bolster the innate defense mechanism by targeting the negatively charged membranes of microorganisms [19]. AMPs have been regarded as a crucial class of natural antibiotics, exerting bactericidal, fungicidal, and virucidal effects, and extensive research has been dedicated to the identification and characterization of AMPs sourced from marine invertebrates, which constitute the largest community of marine inhabitants. The significance of marine invertebrates as a source of antimicrobial molecules lies in the fact that many of these compounds possess unique structures and mechanisms of action and are promising candidates for the development of new antibiotics. In view of the escalating challenge of antibiotic resistance in public health, the quest for novel antimicrobial agents has gained paramount importance [20,21]. Marine invertebrate AMPs offer an advantage in overcoming this challenge, as their mode of action on bacterial membranes makes resistance development more difficult compared to conventional antibiotics.

## 2. Natural Origins and Structural Characteristics of Marine Invertebrate AMPs

To date, over 40 distinct families of AMPs have been characterized in marine invertebrates [22,23]. The structural diversity of these peptides, including various isoforms, is vast [24,25]. Usually, they are divided into five main classes:

(1) Cys-containing peptides stabilized by intramolecular disulfide bonds;
(2) β-hairpin peptides;
(3) Linear α-helical peptides;
(4) Linear peptides enriched in particular amino acid residues (e.g., Gly, Pro, Arg, Trp);
(5) Mixed-type peptides containing domains of different structures.

Brief data on the selected representatives of these families are grouped in Table 1.

The main taxonomic groups of marine invertebrates include Porifera, Cnidaria, Annelida, Mollusca, Echinodermata, Arthropoda, and Chordata. Notably, these taxonomic groups account for two-thirds of all marine animals [68].

### 2.1. Sponges

**Figure 1:**
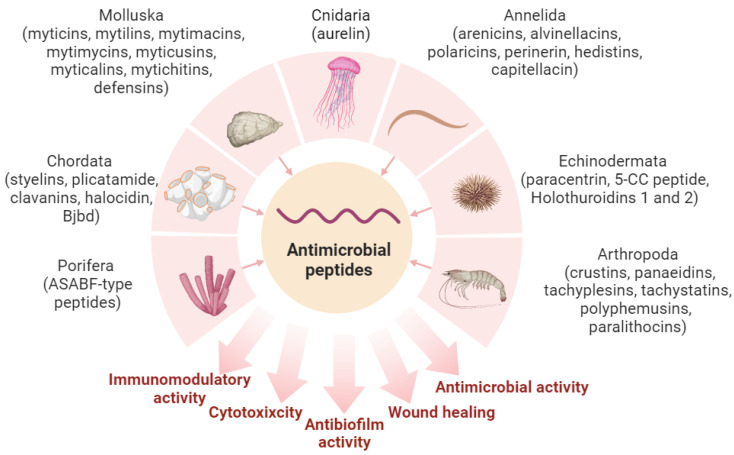
Sources and biological activities of AMPs from marine invertebrates.

Sponges (the phylum Porifera) are the simplest and most primitive marine animals, which possess a unique porous body structure. Sponges hold the distinction of being among the oldest multicellular organisms, with over 8000 described species [69]. Notably, sponges are renowned for their production of a wide array of bioactive compounds. Each year, more than 5000 distinct bioactive substances are identified in sponges [70]. Among marine invertebrates, sponges are considered one of the most abundant sources of AMPs [71]. These peptides exhibit antibacterial, antifungal, and antiviral activities. For instance, the demosponge *Suberites domuncula* produces the peptide of the ASABF type, consisting of 64 amino acid residues, sharing significant similarity with nematode ASABFs (*Ascaris suum* antibacterial factor-type peptides) and having a distant relation to defensins. This peptide exhibits antibacterial and antifungal properties; in addition, it has been shown to lyse human erythrocytes (Figure 1) [72].

### 2.2. Cnidaria

Cnidaria is the phylum containing over 12,000 species of aquatic animals, predominantly marine invertebrates (corals, jelly fish, sea anemones, hydra), which are more complex than sponges [73]. A recent analysis of the mitochondrial genes of cnidarians estimated their age to be around 741 million years old [74]. Aurelin, the 40-residue peptide, exhibiting activity against Gram-positive and Gram-negative bacteria, was purified from the mesoglea of a scyphoid jellyfish *Aurelia aurita*. Aurelin contains six cysteines and has no structural homology with any previously identified AMPs, but reveals partial similarity both with defensins and K+ channel-blocking toxins of sea anemones and belongs to the ShKT domain family [27]. It is known that all members of the phylum Cnidaria are venomous [75]. Aurelin represents a compact globule, enclosing one 3_10_-helix and two α-helical regions cross-linked by three disulfide bonds. The peptide binds to anionic lipid (POPC/DOPG) vesicles even at physiological salt concentration; it does not interact with zwitterionic (POPC) vesicles and interacts with the DPC micelle surface with a moderate affinity via two α-helical regions. Despite the structural homology of aurelin to the BgK and ShK toxins of sea anemones, its surface does not possess the ‘‘functional dyad’’ required for a high-affinity interaction with K^+^ channels [28].

### 2.3. Annelida

The phylum Annelida contains over 13,000 species [73], including polychaeta, which are considered as the most primitive annelids [76]. Many polychaeta species inhabit all oceans and seas—from the Arctic to the Antarctic. Marine polychaetes have been found to produce several types of AMPs, including arenicins [40,41,42,43], capitellacins [48,49], alvinellacin [50], abarenicin [51], UuBRI-21 [51], hedistin [53,54], nicomicins [58], polaricin [60], and perinerin [76]. Each of these AMPs exhibits optimal bactericidal activity against the bacteria commonly found in the habitat of the respective worm species [60]. Arenicins from the lugworm *Arenicola marina* discovered in 2004 [40] have shown significant therapeutic potential [77,78]. Arenicins have demonstrated inhibitory effects on Gram-negative bacteria such as *Escherichia coli* and *Proteus mirabilis*, as well as on Gram-positive bacteria, for example, on *Staphylococcus aureus* [79,80]. Furthermore, arenicin has been shown to modulate the human complement system. At relatively low concentrations, the peptide stimulates complement activation, whereas at higher concentrations, arenicin acts as a complement inhibitor [81,82]. Capitellacins, derived from the marine polychaeta *Capitella teleta,* exhibit a potent antimicrobial activity against a wide range of bacteria, including extensively drug-resistant strains, while showing a low cytotoxicity toward human cells [48]. Perinerin, an antimicrobial peptide isolated from the clamworm *Perinereis aibuhitensis grube*, displays antibacterial activity against both Gram-positive and Gram-negative bacteria, as well as against fungi [77]. Hedistin, isolated from the marine polychaeta *Hediste diversicolor,* is active against Gram-positive bacteria *Micrococcus luteus*, *M. nishinomiyaensis*, and *S. aureus* as well as against the Gram-negative bacterium *Vibrio alginolyticus* [53,83,84]. Hedistin is constitutively produced by NK-like cells circulating in the body cavity of annelids [53]. Nicomicins, isolated from the marine polychaeta *Nicomache minor*, exhibited in vitro antimicrobial activity and possessed cytotoxicity against cancer cells [58]. 

### 2.4. Mollusca

Mollusca is the second most numerous phylum, after Artropoda, among invertebrates. The number of known species exceeds 134,000 [73]. Mollusks, including clams, oysters, and mussels, are recognized for their ability to produce AMPs, possessing inhibitory effects against bacteria and fungi. Among mussels, an astonishing variety of AMPs have been identified, including defensins, big defensins, myticins, mytilins, mytimacins, mytimycins, myticusins, mytichitins, and myticalins [8,29,32,34,35,36,83,84,85,86,87,88,89]. These AMPs are constitutively expressed in various tissues, particularly in the circulating hemocytes [90]. Their precursors typically contain *N*-terminal signal peptides, and mature AMPs usually have total positive net charge and a high proportion of cysteine [87]. Several putative activities have been attributed to mussel-derived AMPs. A plasma peptidomic profiling of the mollusk *Crassostrea hongkongensis* revealed thirty-five peptides, including six up-regulated peptides (URPs). One of them, designated as URP20, exhibited a significant antibacterial activity against the Gram-negative bacterium *Vibrio parahaemolyticus*. The peptide induced bacterial cell aggregation and membrane permeabilization. URP20 also displayed an antibacterial activity against Gram-positive and Gram-negative foodborne pathogens, as well as against *Candida albicans*, without showing cytotoxicity to mammalian cells [91]. Myticin C, expressed constitutively in mussel hemocytes and stored in cytoplasmic vesicles, has demonstrated an antibacterial activity [92] and antiviral effects against fish rhabdovirus, ostreid herpesvirus (OsHV-1), as well as against human herpes simplex viruses HSV-1 and HSV-2 [93,94]. Defensins from the oyster *Crassostrea gigas*, primarily active against Gram-positive bacteria, bind to lipid II, the precursor of peptidoglycan [95]. The oyster bactericidal/permeability-increasing protein (BPI), mainly effective against Gram-negative bacteria, binds to lipopolysaccharide (LPS) [96], which is a component of the cell wall of all Gram-negative bacteria [97,98,99]. Myticusin-beta, derived from the marine bivalve *Mytilus coruscus*, exhibits antibacterial activity against both Gram-positive (*Bacillus cereus*, *B. subtilis*, *Clostridium perfringens*, *S. aureus*, *Streptococcus iniae*) and Gram-negative bacteria (*Escherichia coli*, *Pseudomonas aeruginosa*, *Vibrio alginolyticus*, *Klebsiella pneumoniae*). The peptide also demonstrates antiprotozoal activity [33].

### 2.5. Echinodermata

Members of the phylum Echinodermata, such as starfish, see cucumbers, and sea urchins, have also been found to produce AMPs with a potent antimicrobial activity. The 5-CC peptide derived from the sea urchin *Paracentrotus lividus* has been found to exhibit antibiofilm properties against biofilms formed by *S. epidermidis* and *S. aureus* strains. Interestingly, the sequence of the 5-CC peptide matches the fragment 9–41 of beta-thymosin from *P. lividus* [100]. The peptide paracentrin 1 (SP1) has been chemically synthesized based on the sea urchin beta-thymosin fragment sequence. SP1 demonstrated antibacterial and antibiofilm activities against *S. aureus* and *P. aeruginosa* [101]. The sea cucumber *Holothuria tubulosa* produces AMPs called holothuroidin 1 (H1) and holothuroidin 2 (H2). These peptides display antimicrobial activities against both free-living and biofilm forms of Gram-negative and Gram-positive human pathogens. H1 and H2 are cationic peptides, consisting of 12 and 14 amino acid residues, respectively. They share the same amino acid sequence, except for two additional residues (alanine and serine) at the N-terminus of H2. Both peptides effectively inhibit biofilms formed by staphylococcal and *P. aeruginosa* strains [102].

### 2.6. Arthropoda

#### 2.6.1. Crustacea

Members of the subphylum Crustacea of the phylum Arthropoda, including crabs, shrimps, and lobsters, have been found to produce various AMPs, such as arasin-1, hyastatin, callinectin, hyastatins, crustins, penaeidins, anti-lipopolysaccharide factors (ALFs), and stylicins [63,64,65,66,103]. Penaeidins are small peptides with molecular masses of 5–7 kDa. Each of their precursors consists of an *N*-terminal signal peptide region, a proline-rich domain (PRD), and a *C*-terminal cysteine-rich domain (CRD) containing six cysteine residues. Penaeidins exhibit activity against both Gram-positive and Gram-negative bacteria [67,104,105]. Crustin, initially discovered in the hemolymph of the coastal crab *Carcinus maenas* in 1999, has been subsequently found in other crabs, lobsters, and shrimps [66,106,107,108,109,110,111,112,113]. Crustins are classified into four sub-groups based on their structure and functional activities [66]. They possess antimicrobial activity against Gram-positive bacteria, Gram-negative bacteria, fungi, and viruses [106,114,115,116,117]. Some crustins specifically target Gram-negative bacteria such as *E. coli*, *Edwardsiella tarda*, and *A. hydrophila* [118,119], while others exhibit antibacterial activity against Gram-positive bacteria such as *S. aureus*, *Micrococcus luteus*, and *B. subtilis* [106,120,121,122]. Knockdown of type II crustins has been shown to increase mortality in animals infected with the bacterial pathogen *V. penaeicida*, whereas there was no effect in response to the fungal pathogen *Fusarium oxysporum* [123]. Crustins also possess alternative properties such as agglutination, opsonization, and inhibition of protease activity, further confirming their diverse functions in anti-infective defense [124,125,126,127,128,129]. A hemocyanin-derived peptide, isolated from the penaeid shrimp *Litopenaeus vannamei* and designated as PvHCt, has been shown to be strictly antifungal, causing the permeabilization of the fungal plasma membrane [130]. Paralithocins, derived from the red king crab *Paralithodes camtschaticus*, do not display an antimicrobial activity against *E. coli*, *P. aeruginosa*, or *S. aureus*, but they inhibit the growth of several marine bacterial strains [131].

#### 2.6.2. Chelicerata

Another subphylum Chelicerata of the phylum Arthropoda includes horseshoe crabs, sea spiders, sea scorpions, etc. AMPs from the American horseshoe crab, *Limulus polyphemus*, have been found to bind to LPS and exhibit activity against Gram-negative bacteria [132]. Polyphemusin III, derived from the horseshoe crab *Limulus polyphemus*, has shown a lower antimicrobial effect but a significantly higher cytotoxicity against human cancer and transformed cells in vitro. It has been observed to induce the fast permeabilization of the cytoplasmic membrane of human leukemia cells HL-60, leading to cell death that is apparently unrelated to apoptosis [133]. Tachystatins A, B, C, and tachyplesins have been identified from the hemocytes of the horseshoe crab *Tachypleus tridentatus*. Tachystatins exhibit a broad spectrum of antimicrobial activity against Gram-negative bacteria, Gram-positive bacteria, and fungi [37,38,134,135,136]. Tachyplesins, in addition to their antimicrobial activity, exerts a cytotoxic activity toward human cancer cells, including non-small-cell lung cancer cells, by inducing apoptosis [137,138,139]. Tachyplesins enhance the chemosensitivity of cancer cells to cisplatin, reducing the active concentrations of cisplatin [138,139]. 

### 2.7. Chordata

#### 2.7.1. Tunicata

Members of the subphylum Tunicata of the phylum Chordata, commonly known as tunicates, represent another significant source of AMPs. Among these are ascidians (sea squirts), sea tulips, sea pork, sea livers, etc. A group of peptides, termed styelins, has been identified in the ascidian tunicate *Styela clava* [57,58,140]. Styelins share a close resemblance to the antimicrobial peptide cecropin found in the hemolymph of the silkworm caterpillar *Hyalophora cecropia* [141]. These styelins exhibit antimicrobial activity against both Gram-positive and Gram-negative bacteria and also possess hemolytic and cytotoxic properties toward eukaryotic cells [58,142]. Halocidin, derived from hemocytes of the tunicate *Halocynthia aurantium*, demonstrates antifungal activity against the *Candida* species, including *Candida*-related oral infections [53,143,144]. Furthermore, halocidin congeners, known as Khal, exhibit potent antibacterial effects against methicillin-resistant *S. aureus* (MRSA), vancomycin-resistant Enterococcus (VRE), and various multidrug-resistant strains of *P. aeruginosa*. Halocidin analogs have shown promising results in animal models of *Listeria monocytogenes* infection [145]. Clavanin A, isolated from the hemocytes of the sea squirt *Styela clava*, exhibits a robust antibacterial activity against Gram-positive bacteria, including *Enterococcus faecium* and methicillin-resistant *S. aureus* strains, as well as against Gram-negative bacteria, such as *E. coli* and *P. aeruginosa* [57,146,147,148,149,150]. Plicatamide, derived from hemocytes of the sea squirt *Styela plicata*, demonstrates an antibacterial activity against methicillin-resistant strains of *S. aureus* (MRSA) and a hemolytic activity toward human erythrocytes [151]. Tunicates also serve as a source for peptides known as didemnins, which possess antimicrobial activity against various pathogens and demonstrate cytotoxicity toward human cancer cells [152,153,154,155,156,157].

#### 2.7.2. Cephalochordata

Another subphylum Cephalochordata of the phylum Chordata is represented by amphioxus, also known as lancelet. Amphioxus and tunicates, being the most primitive chordates, belong to invertebrates. AMPs structurally related to large defensins from the the amphioxus *Branchiostoma japonicum* (designated as Bjbd) have been identified in databases using signal and propeptide sequences, which are typically significantly more conserved than those of mature peptides [30,158]. The mature large defensin consists of 117 amino acid residues and has the hydrophobic region GAAAVT(A)AA at the N-terminus and the consensus pattern C-X6-C-X3-C-X13(14)-C-X4-CC at the C-terminus, as well as four α-helices, four β-sheets, and three disulfide bridges (C1-C5, C2-C4, and C3-C6). Quantitative real-time PCR analysis has revealed that Bjbd was constitutively expressed in most tissues examined, and its expression significantly increased following incubation with LPS or LTAs, as well as upon infection with *Aeromonas hydrophila* or *S. aureus*. Moreover, the recombinant BjBD has been shown to inhibit the growth of *S. aureus*, *E. coli,* and *A. hydrophila* [30]. This was the first large defensin gene ever identified in the Chordata phylum. Thus, in silico approach integrating experimental study has revealed the existence of the novel AMP and has allowed investigators to define its biological functions [30,158,159]. Several AMPs have been designed by amino acid substitutions in the peptide mBjAMP1 isolated from *Branchiostoma japonicum*, and their activities have been tested. It has been founded that some analogs had the ability to kill Gram-negative *Vibrio anguillarum*, *Pseudomonas mendocina*, *Vibrio parahaemolyticus*, and Gram-positive *Micrococcus luteus* and *Listeria monocytogenes*. Additionally, all the four AMPs induced the permeabilization and depolarization of bacterial cell membranes, increased intracellular reactive oxygen species (ROS) levels, and had little or no mammalian cytotoxicity [159]. Other analogs of the Bjbd peptide designated as ARR-Anal10 displayed not only the greatest antimicrobial and antibiofilm activities, but also no toxicity toward human red blood cells or other mammalian cells. IARR-Anal10 had little or no effect on bacterial outer membrane permeability, membrane polarization, or membrane integrity. Instead, IARR-Anal10 binds to bacterial DNA and kills bacteria through an intracellular mechanism. It has been confirmed that IARR-Anal10 suppressed the virulence of *K. pneumoniae* to a degree similar to tigecycline, used to treat carbapenem-resistant *Enterobacteriaceae* infections, and did not induce the development of resistance by *K. pneumoniae* [160]. Notably, AMPs from amphioxus were discovered using bioinformatics and systems biology, which integrate research data and serve as a basis for drug design and novel AMPs [161,162]. It was found that the amphioxus ribosomal polypeptide RPS23, designated as BjRPS23, acted not only as a pattern recognition receptor (PRR) capable of identifying LPS, LTAs, and PGN, but also as an effector killing Gram-negative and Gram-positive bacteria. BjRPS23 functions through the combined membrane-lytic mechanism, including interaction with the LPS, LTAs, and PGN of the bacterial membrane, as well as by membrane depolarization. BjRPS23 also stimulates the production of intracellular ROS in bacteria and is not cytotoxic to mammalian cells, thereby being a promising molecule for the development of new peptide antibiotics against bacteria [163].

## 3. Biosynthesis and Gene Expression Regulation of AMPs in Marine Invertebrates

**Figure 2:**
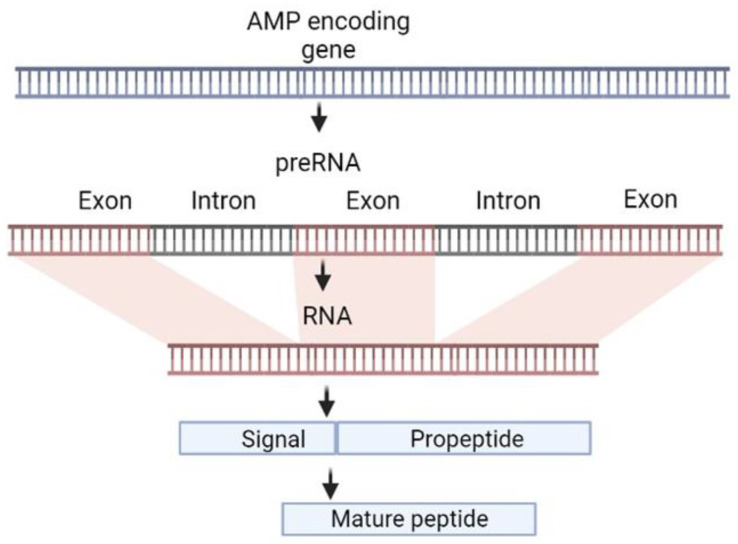
Biosynthesis of gene-encoded AMPs.

The biosynthesis of AMPs in animals involves the transcription and subsequent translation of the genes of the corresponding precursor proteins, containing the *N*-terminal secretory signal sequence [164]. Sometimes, AMP precursors feature a prodomain (or propeptide) positioned between the signal and mature peptide sequences or occupying the molecule’s *C*-terminal region. These prodomains usually have chaperone-like properties that counteract the membrane activity and aggregation propensity of the mature peptide, protecting the producer organism from its action (Figure 2). Some of them are short anionic polypeptides (15–30 amino acid residues) that neutralize the positive charge of mature peptides, such as the propeptides of centrocins and strongylocins of sea urchins [165]. Another example is BRICHOS, a sequence of about 100 residues found within the precursors of the β-hairpin AMPs of marine polychaetes: arenicins, alvinellacin, nicomicins, capitellacin, and abarenicins [40,48,49,50,51]. The BRICHOS domain, which has also been found in several human proteins, displays a notable degree of evolutionary conservation at the level of overall domain architecture, while the primary structure undergoes significant changes [166]. A distinct group of AMPs are “encrypted” peptides, which are parts of larger proteins that have an independent physiological function. They are formed as a result of partial proteolysis of these proteins and play an important role in host immunity. The best known representatives of this group of AMPs are histone derivatives from fish and amphibians, but in marine invertebrates, hemocyanin is the main source of known peptides of this group [167,168,169].

The signal peptide directs the precursor protein to the endoplasmic reticulum (ER), where it undergoes processing and, in some cases, post-translational modifications. Finally, the mature AMPs are transported to the site of infection, where they exert their antimicrobial activity by disrupting microbial membranes or interfering with intracellular processes.

The genes of AMP precursors are often found in the genomes in clusters encoding natural libraries of bioactive peptides. For example, genes for 37 antimicrobial peptides were identified in the American lobster *Homarus americanus* genome, including nine ALFs, 23 crustins/carcinins, and five β-defensin-like panusins [170]. The evolution of gene clusters involves such events as gene duplication, changes in gene copy number, recombination, and allelic polymorphisms, although the evolutionary factors responsible for shaping the diversity of AMPs remain mostly unknown [171]. The expression of AMPs is highly variable in tissues, with some being produced constitutively, accumulating in granules of immune system cells, whereas the synthesis of others is induced by pathogens. A number of studies have reported that AMP genes exhibit different expression patterns in response to different environmental stimuli and conditions such as microorganism species, type of pathogen-associated molecules, the temperature of the environment, and the developmental stage of the organism [67,172,173,174].

When a microbe infects an invertebrate, pattern recognition receptors (PRRs) on the surface of immune cells recognize conservative pathogen-associated molecular patterns (PAMPs) displayed by the invading microorganism [9,10]. These include bacterial peptidoglycans, LPS, teichoic acids, and flagellin; fungal mannans, glucans, chitin, and ergosterols; and double-stranded RNA of viruses. This recognition event triggers a signaling cascade that activates transcription factors. These transcription factors then bind to the promoter region of AMP genes, initiating the process of transcription [66]. The regulation mechanisms of invertebrates’ immune response have been extensively studied in the model organism *Drosophila melanogaster* [175]. The main signaling pathways providing the induction of the synthesis of protective factors in this insect are the Toll and Imd pathways, which activate two NF-κB transcription factors, Dif/Dorsal and Relish, respectively. This in turn leads to the activation of κB-containing promoters of genes associated with the innate immune system. A similar regulatory system was found in cultured shrimps, crabs, crayfish, and lobsters whose host defense mechanisms have been deeply investigated over the last 20 years due to the high importance of these crustaceans to the economies of Southeast Asian countries [176,177]. Numerous homologs of proteins responsible for PAMP recognition and signal transduction in *Drosophila* were found in marine crustaceans: Toll [178] and Imd [179] receptors, Spätzle [180], MyD88 [181], Tube [182], Pelle [183], Dorsal [184], Relish [185], and others. However, the mechanisms of signal transduction may differ from those observed in insects. For example, in contrast to the *Drosophila* Toll, which recognizes PAMPs through binding to Spätzle, shrimp Tolls, similar to mammalian counterparts, can directly bind to the pathogen-associated motifs [186]. While *Drosophila* Toll signaling pathway is activated by Gram-positive bacteria and fungi, and IMD is activated by Gram-negative bacteria [187], no such specialization of the two signaling systems was found in shrimp [169]. Gene expression of penaeidins, crustins, and ALFs can be activated through both signaling pathways. In addition, some other signaling pathways, such as JAK-STAT, may be involved in the regulation of the biosynthesis of crustins and ALFs [188].

Thus, the regulation of AMP gene expression is a complex process involving the interplay of multiple signaling pathways and transcription factors. It was found that out of the several dozen AMP genes that may be present in the genome, only a few show high expression levels, e.g., a transcriptome profiling of the red king crab *Paralithodes camtschaticus* revealed that among 27 AMP genes, only paralithocin 1 and crustin 3 genes yield a physiologically relevant amount of expression product [189]. The conservation of natural peptide libraries during phylogeny fits well with the “screening hypothesis”: the ability to cost-effectively generate diverse libraries of metabolites (only a small fraction of which will be active under given habitat conditions) is an important evolutionary advantage that allows a species to readily occupy new ecological niches [190].

The expression of AMP genes in marine invertebrates is regulated not only by microbial infection, but also by the developmental stage and environmental cues such as temperature, pH, and nutrient availability [60,191]. Some AMPs are specifically expressed during certain developmental stages, while others are induced in response to specific environmental signals [60]. For example, crustins from penaeid shrimps *Litopenaeus vannamei* have been detected in all stages of development, from fertilized eggs to larval and postlarval stages [65].

In summary, the biosynthesis and regulation of the gene expression of AMPs in marine invertebrates are complex and dynamic processes that enable these organisms to effectively defend against microbial infections of different etiologies during all stages of ontogenesis and under various environmental conditions [59]. 

## 4. Structural Characteristics of AMPs in Marine Invertebrates

**Figure 3:**
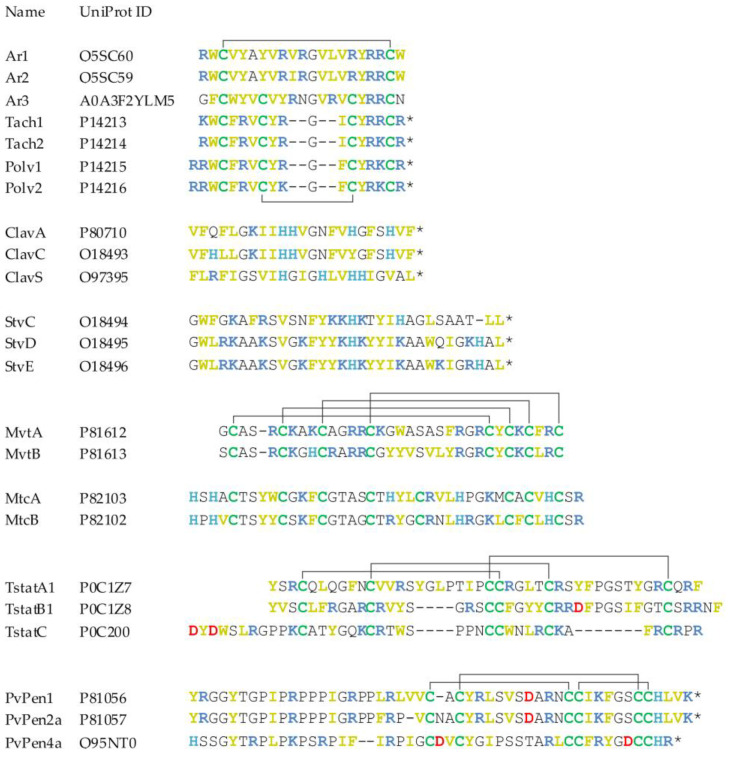
Amino acid sequence alignments of several families of marine invertebrate HDPs. Ar1, Ar2, Ar3—arenicins-1, -2, -3 from *Arenicola marina* (lugworm); Tach1, Tach2—tachyplesins I and II from *Tachypleus tridentatus* (Japanese horseshoe crab); Poly1, Poly2—polyphemusins 1 and 2 from *Limulus polyphemus* (Atlantic horseshoe crab); ClavA, ClavC, ClavS, StyC, StyD, StyE—clavanins A, C, clavaspirin, and styelins C, D, E from *Styela clava* (Sea squirt); MytA, MytB—mytilins A and B from *Mytilus edulis* (Blue mussel); MtcA, MtcB—myticins A and B from *Mytilus galloprovincialis* (Mediterranean mussel); TstatA1, TstatB1, TstatC—tachystatins from *T. tridentatus*; PvPen1, PvPen2a, PvPen4a—penaeidins 1, 2a, 4a from *Penaeus vannamei* (Whiteleg shrimp). Basic residues (HKR) are shown in blue; acidic (D)—in red; highly hydrophobic (FILVWY)—in yellow; cysteine residues—in green. The arrangement of disulfide bonds and the amidated *C*-terminal residues (*) are shown. Other modifications, such as hydroxylated and brominated residues, are not shown in the figure.

**Figure 4:**
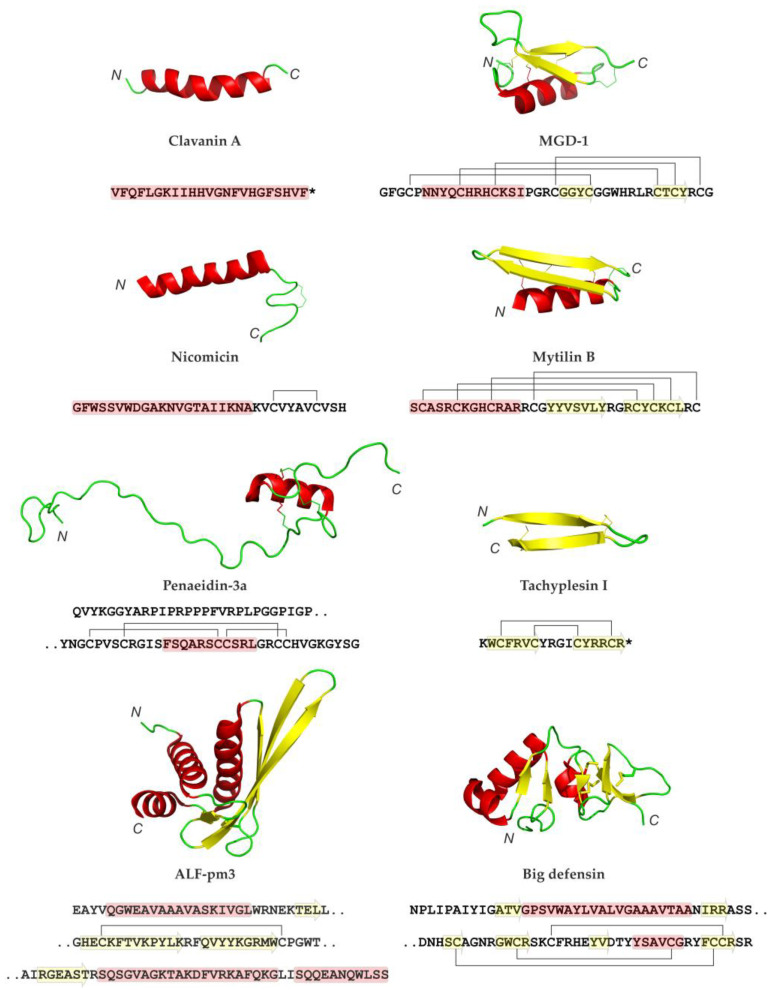
Primary and NMR solution structure of representative AMPs of marine invertebrates: clavanin A (PDB 6C41) from the tunicate *Styela clava*; defensin MGD-1 (PDB 1FJN) and mytilin B (PDB 2EEM) from the mussel *Mytilus galloprovincialis*; nicomicin (PDB 6HN9) from the polychaeta *Nicomache minor*; penaeidin-3 (PDB 1UEO) from the shrimp *Litopenaeus vannamei*; tachyplesin I (PDB 1WO0) and “big defensin” (PDB 2RNG) from the horseshoe crab *Tachypleus tridentatus*; anti-LPS factor ALF-Pm3 (PDB 2JOB) from the shrimp *Penaeus monodon*; *N*- and *C*-terminal residues are labeled, amidated *C*-terminal residues are shown with an asterisk. The regions of the primary structures adopting the α-helical and β-sheet conformation are highlighted in red and yellow, respectively.

AMPs of marine invertebrates exhibit a remarkable diversity in terms of structure, charge, and hydrophobicity profiles, which contributes to their broad-spectrum protection against various pathogens (Figure 3 and Figure 4). Most of them share common features of membranotropic AMP: they are enriched in lysine and arginine residues, which provide them with a positive charge, and also contain a high proportion of hydrophobic residues. The spatial segregation and clusterization of hydrophobic and hydrophilic residues often observed in these peptides provides them with pronounced amphiphilic properties and an affinity to biological membranes.

The molecules of marine invertebrate AMPs are composed mostly of unmodified residues of proteinogenic L-amino acids. The most common type of post-translational modification is the formation of disulfide bonds that stabilize the spatial structure of cystein-containing peptides. Intramolecular disulfide bonds increase the resistance of peptides to proteolysis in physiological media. In some cases, their presence is a prerequisite for antimicrobial activity. For example, the reduced myticin C loses its antibacterial activity at neutral pH values, although it retains chemotactic properties [192]. In contrast, for beta-hairpin AMPs, such as arenicins and tachyplesins, disulfide bonds do not play such a crucial role [135,193,194]. The stabilization of these molecules can be achieved by hydrophobic and cation–π interactions between the opposite residues of two β-strands [193].

Another type of a post-translational modification of AMPs is the amidation of the *C*-terminal amino acid residue catalyzed by a bifunctional enzyme: peptidylglycine α-amidating monooxygenase [195]. The peptide precursor must thus contain an additional *C*-terminal glycine residue, which is oxidized during the reaction. The amidation of the *C*-terminal residue is found, in particular, in β-hairpin peptides from horseshoe crab hemocytes, tachyplesins, and polyphemusins [45]. This modification increases the net positive charge of the molecule by +1, increasing its affinity for negatively charged components of microbial membranes. In addition, it increases the half-life of the peptide in biological media, making it resistant to the action of carboxypeptidases [196]. The *N*-terminal glutamine residue can be cyclized to form pyrrolidone carboxylic (pyroglutamic or pGlu) acid, as observed in peneidin-3a from the whiteleg shrimp *Penaeus vannamei* [197] and the light chain of EeCentrocin 2 from sea urchin *Echinus esculentus* [198]. Some marine AMPs contain hydroxytryptophan [86], dihydroxylysin, dihydroxyarginine, and dihydroxyphenylalanine [57]. A modification highly specific to marine peptides is the bromination of tryptophan residues observed in centrocins and strongylocins [59,165,198], styelin D [57], and hedistin [53]. These modifications generally do not affect antimicrobial activity and are likely intended to enhance the proteolytic resistance of the peptides [24]. At least one exception is known: a synthetic analog of stiellin D, which does not contain modifications, loses the salt tolerance inherent in the natural peptide [57].

The structure diversity of marine invertebrate AMPs was previously classified into four main types, as proposed by Semreen and Bertrand: peptides adopting a β-hairpin or helical/β-sheet structure stabilized by intramolecular disulfide bonding (cyclic peptides), linear α-helical peptides, and helical or non-helical peptides with an abundance of a specific amino acid [23,199]. This list can be supplemented by a group of mixed-type peptides containing domains of different structures, such as arasin 1, crustins, and penaeidins. With regard to the general structural organization of peptide molecules, it is worth noting that, compared to land animals, the AMP repertoire of marine invertebrates is characterized by a higher content of cysteine. The proportion of linear molecules that do not contain disulfide-bonded rings is relatively small. Some examples of linear α-helical peptides are hedistin from polychaeta *Nereis diversicolor*, clavanins, styelins, and clavaspirin from ascidian *Styela clava*. Dicyntharin and halocidin from ascidian *Halocynthia aurantium* [52,55] and centrocins from sea urchins [165], which are formally assigned to the group of linear helical peptides, are unusual in that they are formed by combining fragments of different polypeptide chains via single disulfide bonding.

Defensins are the most widely distributed and conserved cysteine-containing AMPs found in all kingdoms of eukaryotic organisms. The first classical defensin isolated from marine invertebrates was MGD-1 from the mussel *Mytilus galloprovincialis* [200]. The three-dimensional solution structure of MGD-1 established using NMR consists of an α-helical *N*-terminal part and two antiparallel β-strands forming a common cystine-stabilized αβ-motif (CSαβ) often found in scorpion toxins and defensins of Chelicerata and ancient orders of insects. The pattern of cysteine residues, as well as the arrangement of disulfide bonds and secondary structure, distinguish invertebrate defensins from vertebrate α- and β-defensins. Unlike most animal defensins, MGD-1 contains eight (instead of six) cysteine residues and forms four intramolecular disulfide bonds—this structural feature makes it similar to plant defensins. Defensin-like AMPs include mytilins from mussel and “big defensins” found in hemocytes of the horseshoe crab *Tachypleus tridentatus*, several species of bivalve mollusks, and a lancelet [29,30,201,202]. “Big defensin” from *T. tridentatus* consists of two functional domains with different spectra of antimicrobial activity encoded by two separate exons. According to NMR data, the more hydrophobic and weakly basic *N*-terminal domain forms a globule in solution, while in the lipid environment, it adopts an elongated α-helical conformation [203]. The *C*-terminal domain is similar to vertebrate β-defensins in terms of disulfide bond arrangement and spatial structure, which suggests their evolutionary relationship [204].

As has been shown for CSαβ-containing peptides, the same molecular scaffold can be found in peptides with different biological activities. Conserved cysteine motifs present in AMPs are sometimes identified in peptides and protein domains belonging to other functional classes. Another example, aurelin from the mesoglea of the *Aurelia aurita* jellyfish, shares similarities with potassium channel-blocking toxins from marine anemones at the level of the primary and spatial structure, which includes two crossed α-helices [28].

Among the most active antibiotic molecules of marine animals are β-hairpin peptides—small (~2.5 kDa) peptides formed by two twisted antiparallel β-strands joined by β-turn. Most members of the family (tachyplesins, polyphemusins, alvinellacin, nicomicins, capitellacin, abarenicins) contain two disulfide bonds. The exceptions are arenicins-1 and -2 containing a single disulfide bridge that forms a large 18-residue ring [40].

During the last several years, the intensive study of the innate immunity of decapod crustaceans has led to an explosive growth in the number of known representatives of three families of cysteine-containing AMPs: ALFs, crustins, and penaeidins. ALFs are small proteins with a molecular mass of 10–12 kDa, containing three α-helices adjacent to a β-folded region consisting of four β-strands [205]. The second and third β-strands are connected by the only disulfide bond present in the molecule, forming an LPS-binding domain enriched with charged amino acid residues. ALFs have an amphiphilic structure with a strongly hydrophobic *N*-terminal region. Although some ALFs are anionic peptides, antimicrobial activity has been found only in those members of the family that have a net positive charge.

Penaeidins, AMPs from shrimp hemocytes, consist of a proline-rich *N*-terminal part and a conserved *C*-terminal fragment containing six cysteine residues that form disulfide bonds in the order 1–3, 2–5, 4–6. The middle part of the *C*-terminal domain has the conformation of an amphiphilic α-helix closely bound to the preceding and following regions of the polypeptide chain [206]. There is a similarity between the primary structure of the *C*-terminal fragment of penaeidins and the chitin-binding domains of plant proteins.

Compared to other AMPs, crustins have a more complex domain architecture. The first member to be discovered was carcinin (crustin Cm1), isolated from the hemolymph of the green crab Carcinus maenas. The peptide has a molecular mass of 11.5 kDa and contains 12 cysteine residues [207]. Common to all crustins is a cationic *C*-terminal WAP domain (Whey Acidic Protein) containing twelve cysteine residues. The WAP domain has previously been found in mammals as part of some protease inhibitors as well as antimicrobial proteins. The *N*-terminal region of the crustin molecule may contain glycine-rich, proline/arginine-rich, or cysteine-rich domains, a second WAP domain, or a region enriched in aromatic amino acids. The domain composition of the molecule became the basis for the classification of crustins, according to which they are subdivided into seven groups [208].

Here, we have briefly summarized the main features of only some of the most representative and well-studied families among the great diversity of marine invertebrate AMPs. At the same time, it is clear that only a fraction of the AMPs present in marine invertebrates have been identified thus far, indicating that these organisms hold great potential as a vast resource for researchers to discover biologically active molecules [11,23,209,210].

## 5. Spectrum of Biological Activities of Marine Invertebrate AMPs

AMPs derived from marine invertebrates exhibit a broad spectrum of biological activities, making them important components of the innate immune system. They possess antibacterial, antifungal, and antiviral properties, collectively contributing to the defense against various pathogens encountered in marine environments. An antibacterial activity of AMPs enables them to target and eliminate a wide range of bacterial species, including both Gram-positive and Gram-negative bacteria. By disrupting the integrity of bacterial cell membranes or interfering with intracellular targets, AMPs can effectively inhibit bacterial growth and proliferation. Furthermore, AMPs from marine invertebrates display potent antifungal activity, enabling them to combat fungal pathogens. These peptides can disrupt fungal cell membranes, inhibit fungal growth, and prevent the spread of fungal infections. Additionally, certain AMPs derived from marine invertebrates possess antiviral properties, allowing them to directly target and inhibit the replication of viruses. They can interfere with viral entry into host cells, disrupt viral envelopes, or inhibit viral protein synthesis, thus exerting antiviral effects.

The diverse biological activities of AMPs from marine invertebrates underline their crucial role in the innate immune defense of these organisms. By providing protection against bacterial, fungal, and viral infections, these peptides contribute to the overall health and survival of marine invertebrate species in their natural habitats. Given below are some examples of biological activities of marine invertebrate AMPs.

### 5.1. Antibacterial Activity

AMPs derived from marine invertebrates exhibit a broad-spectrum antibacterial activity against both Gram-positive and Gram-negative bacteria (Table 1). Some AMPs display a specific activity, while others possess antibacterial properties against both types of bacteria. For instance, arenicins from the marine polychaeta *Arenicola marina* and halocidin from the tunicate *Halocynthia aurantium* have demonstrated effectiveness against Gram-positive bacteria *S. aureus*, *B. subtilis*, and *L. monocytogenes*, as well as against Gram-negative pathogens *E. coli* and *P. aeruginosa* [40,211,212,213]. Marine mollusk-derived AMPs, such as mytilins, myticins, mytimacin, mytimycin, myticusins, mytichitins, myticalins, and big defensin, have putative antibacterial activity against both Gram-positive and Gram-negative bacteria [8,9,32,36,87,88,89]. It is worth noting that marine organisms possess a diverse variety of AMPs that specifically target harmful bacteria prevalent in their respective environments. Antibiofilm activities of abarenicin from the polychaete *Abarenicola pacifica*, paracentrin 1 and 5-CC peptides from the sea urchin *Paracentrotus lividus*, and holothuroidin from the sea cucumber *Holothuria tubulosa* predetermine their future topical application [51,100,101,102,103].

### 5.2. Antifungal Activity

AMPs derived from marine invertebrates have demonstrated a significant antifungal activity against various fungal pathogens. Mytimycin from the blue mussel *Mytilus edulis* inhibits the growth of *Neurospora crassa* and *Fusarium culmorum* [36,214]. Tachystatins A, B, and C, derived from hemocytes of the horseshoe crab *Tachypleus tridentatus*, have induced morphological changes in budding yeast [37,38]. Halocidin isolated from hemocytes of the *Halocynthia aurantium* sea squirts has shown a pronounced activity against *Candida* even after short-term incubation. Furthermore, it has demonstrated a significant reduction in fungal burden in a mouse model of oral candidiasis, without being absorbed into the bloodstream [144]. The histidine-rich antimicrobial peptide PvHCt derived from the penaeid shrimp *Litopenaeus vannamei* possesses an antifungal activity via selective binding to *Fusarium oxysporum* cells and permeabilizing them [130].

AMPs, which exhibit both antibacterial and antifungal activity, are of particular interest to researchers. A peptidomic profiling of the mollusk *Crassostrea hongkongensis* plasma revealed thirty-five up-regulated peptides (URPs) when infected with the Gram-negative bacterium *Vibrio parahaemolyticus*. The URP20 peptide had a significant antibacterial activity and triggered the aggregation of bacterial cells, accompanied by the permeabilization of their membranes. URP20 has been found to be active against Gram-positive and Gram-negative foodborne pathogens, as well as against *Candida albicans*, with no cytotoxicity to mammalian cells [91,215]. The rEsDWD polypeptide from the Chinese mitten crab *Eriocheir sinensis* has shown an antimicrobial activity against the Gram-negative bacteria *V. anguillarum*, as well as against the yeast *P. pastoris* GS115 strain and *Candida parapsilosis* [117]. These findings highlight the potential of marine invertebrate-derived AMPs as effective antifungal agents with therapeutic implications.

### 5.3. Antiviral Activity

Marine invertebrate AMPs have demonstrated a notable activity against various viruses, including herpes simplex virus, human immunodeficiency virus, influenza virus, hepatitis C virus (HCV), or SARS-CoV-2 [216]. Depsipeptides from the sponges *Theonella mirabilis* has shown an immediate virucidal effect of the human immunodeficiency virus 1 (HIV-1) inhibition through a viral membrane-targeting mechanism resulting in the subsequent viral membrane disruption and viral inactivation [217].

Notably, tachyplesin from the *Tachypleus tridentatus* horseshoe crab has exhibited a potent antiviral activity against herpes simplex virus [218]. Myticin C, derived from hemocytes of the *Mytilus galloprovincialis* mussel, has shown an antiviral action against fish rhabdovirus, ostreid herpesvirus, and human herpes simplex viruses 1 and 2, affecting the intracellular phase of viral replication [92,93].

### 5.4. Cytotoxicity

Structural characteristics of AMPs from marine invertebrates, such as a total positive charge and amphiphilicity, provide the ability to target negatively charged lipid components of bacterial membranes and display a potent cytotoxicity [17]. Mammalian cell membranes contain zwitterionic phospholipids and cholesterol, which are thought to be protected against the effects of AMPs. The selectivity of AMPs toward bacterial cells is an important advantage. However, not all AMPs from marine invertebrates exhibit such a selectivity, and the hemolytic activity and cytotoxicity of AMPs toward human cells are some of the main barriers to their widespread usage. For instance, nicomicins from the polychaeta *Nicomache minor* possess cytotoxicity against cancer cells (HeLa) and human erythrocytes [58].

A search for analogs with a pronounced antibacterial activity and a low cytotoxicity is underway. In particular, a comparison of biological activities of arenicin-1 from the marine polychaete *Arenicola marina* and its analog Ar-1[V8R] revealed that Ar-1[V8R] exhibited a significantly reduced cytotoxicity toward mammalian cells compared to the wild-type arenicin-1 while maintaining the antibacterial activity. Moreover, a comparative NMR analysis of the peptides’ spatial structures showed that Ar-1[V8R], unlike arenicin, has a significantly lower dimerization propensity [43]. On the other hand, the cytotoxicity of AMPs is supposed to be used to combat transformed and virus-infected cells. For instance, the cytotoxicity of tachyplesins toward human cancer cells, including non-small-cell lung cancer and cisplatin-resistant A549/DDP cells, enhances the chemosensitivity to cisplatin and has a promising potential as an antitumor drug [137,138,139]. Carriers and drug delivery methods are being developed to target tumor and HIV-infected cells [217,218].

## 6. Mechanisms of Antimicrobial Action of AMPs in Marine Invertebrates

AMPs derived from invertebrates employ various mechanisms to effectively kill or inhibit the growth of microorganisms. These mechanisms can be broadly categorized into membrane disruption, intracellular targeting, and immune modulation. Several models have been proposed to elucidate the mechanisms of direct action of AMPs, including the barrel-stave model, toroidal pore model, carpet model, aggregation model, molecular electroporation model, and sinking raft model. These models describe different ways in which AMPs can disrupt the cell membrane, which is crucial for the survival of microorganisms [219,220,221]. Regardless of the molecular target and mechanism of action of AMPs, the process begins with the adsorption on the surface of the microorganism cell, provided by the electrostatic attraction of the cationic peptide to the negatively charged components of the membrane and cell wall. An increase in the ionic strength of the solution usually inhibits this interaction and reduces the activity of AMPs. However, a number of marine invertebrate peptides are partially resistant to the presence of salts in the medium [57,207,222,223]. The next step is the insertion of the peptide molecules into the layer of charged phospholipid heads and then into the hydrophobic part of the lipid bilayer. This stage is usually associated with an increase in membrane permeability and a complete or partial loss of its barrier function. The efficiency of membrane lysis is often dependent on the ability of the peptide to aggregate and form oligomeric complexes. Depending on the involvement of lipid molecules, barrel-stave pores (whose inner surface is lined exclusively with peptide molecules) or toroidal pores (lined with charged phospholipid heads alternating with peptide molecules) are formed. In some cases, when high concentrations of peptides are involved, instead of the stable pores formation, a detergent-like lysis through the so-called carpet mechanism occurs. The above mechanisms, proposed at the dawn of the AMP research era, are to a greater or lesser extent common to all families of cationic AMPs possessing an amphiphilic structure or are capable of acquiring such a structure upon contact with the lipid bilayer. However, in each particular case, specific interactions of AMPs with receptor molecules on the surface of the target cell, and sometimes inside it, may occur.

Thus, cysteine-containing AMPs of the defensin family were initially considered to be membrane-active agents that increase the permeability of the lipid bilayer and induce a transmembrane ion current detrimental to the target cell. However, in 2010, it was shown that the main mechanism of action of many defensins is the inhibition of peptidoglycan biosynthesis through the binding to lipid II [222,223,224]. In particular, this is the mechanism of action of three oyster defensins (Cg-Defh1, Cg-Defh2, and Cg-Defm) [95]. In contrast to vertebrate defensins, which have a broad spectrum of activity, invertebrate defensins have no affinity for LPS, but have a higher affinity for lipid II, which explains their selectivity against Gram-positive bacteria. Therefore, antibacterial defensins share a common target with some lantibiotics (such as nisin), the glycopeptide antibiotic vancomycin, and the depsipeptide antibiotic teixobactin. Defensins do not induce cross-resistance with vancomycin, which binds to the D-Ala-D-Ala fragment, since their target is another more conserved site, containing pyrophosphate moiety. The binding is irreversible and occurs in a 1:1 stoichiometric ratio. The key determinants of binding to lipid II are most likely the evolutionary conserved residues Phe-2, Gly-3, Cys-4, and Cys-25 [95]. Antibacterial specificity of different peptide isoforms evolves under diversifying selection by a redistribution of positively charged residues exposed on the surface of the molecule. Remarkably, at concentrations 10 times higher than their MICs, oyster defensins do not cause the depolarization of *Staphylococcus aureus* membrane [95]. However, it cannot be excluded that other members of the defensin family may simultaneously inhibit peptidoglycan synthesis and disrupt membrane permeability.

Penaeidins and AMPs from the horseshoe crab *Tachypleus tridentatus* are known to have the ability to bind chitin. It has been observed that affinity to chitin correlates with antifungal activity: tachyplesins and tachystatins, which bind to chitin more strongly than “big defensin” and tachycytin, show their effect against *Candida albicans* and *Pichia pastoris* at much lower concentrations [37]. Since the animals producing these AMPs are themselves covered with a chitin shell, it is possible that they not only provide protection against fungal pathogens, but also participate in the assembly and regeneration of the chitin exoskeleton [225].

For many cationic peptides which penetrate microbial cells, nucleic acids may be a potential target. Binding to them inhibits replication and transcription processes. Although the main mechanism of action of the β-hairpin AMP tachyplesin I is currently considered to be membrane depolarization due to the formation of toroidal pores [226], one of the first reports showed its ability to bind to the minor groove of DNA [227]. According to more recent studies, tachyplesin may kill bacteria by targeting intracellular enzymes [228], particularly FabG, the conserved 3-ketoacyl carrier protein reductase in the unsaturated fatty acid biosynthesis pathway [229]. Another well-known target of this versatile AMP is lipopolysaccharide (LPS) of Gram-negative bacteria. It was shown via NMR spectroscopy that the majority of positively charged (R4, R7, R11, and R13) and aromatic residues participate in the interaction of the cysteine-deleted analog of tachyplesin-1 with LPS. In particular, there may be ionic interactions between guanidinium groups of residues R7 and R13 with di-phosphate groups of the lipid A moiety [230].

The antibacterial action of arenicin-1, another β-hairpin AMP, has been explained by the oligomerization in the lipid bilayer with the formation of toroidal pores, or by action via a carpet-like mechanism [42,231]. Amino acid substitutions in the non-polar face of the molecule with hydrophilic residues can reduce the propensity of the peptide to dimerize, thereby reducing the hemolytic activity without affecting its antimicrobial activity [43]. The fungicidal action of this peptide against *Candida* is associated with the generation of reactive oxygen species and the induction of apoptosis [232].

The target for cationic ALFs, as their name suggests, is LPS. The main functional region of ALFs is the LPS-binding domain (LPS-BD or LBD)—a β-hairpin, stabilized by the disulfide bridge. The positively charged residues of LPS-BD bind in an exothermic reaction with the negative charges of lipid A moiety, and then the interaction of the β-hairpin with the LPS acyl chains takes place [233,234]. Both the opening of the disulfide bridge and replacing the cationic residues with neutral ones diminished the antibacterial effects [235]. However, the spectrum of activity of these compounds is much broader and also includes Gram-positive bacteria, fungi, and viruses [236]. The mechanism of action of ALFs against non-bacterial pathogens remains to be elucidated, but it has been shown that lipoteichoic acids (LTAs) serve as a molecular target when acting on Gram-positive bacteria. Binding to LPS and LTAs ultimately leads to the destabilization of bacterial membranes. The same is also true for crustins. Crustins bind to cell wall components such as peptidoglycan, LTAs, and LPS. It has been observed that at least two molecules of crustins interact with one LTAs or LPS molecule [105]. Furthermore, the antimicrobial activity of crustins correlates with their specificity for binding to bacterial cell wall components [106].

Summarizing the above, we can conclude that many AMPs seem to exert their antimicrobial action through different parallel mechanisms. The prevalence of one or another mechanism may depend on peptide concentration and environmental conditions. This makes it very difficult to identify the causes of death or the growth inhibition of the target microorganism.

## 7. Marine Invertebrate AMPs as Molecular Factors of Innate Immunity

AMPs derived from marine invertebrates play a vital role in their innate immune system, which provides a rapid and non-specific response to invading pathogens. Produced by various cell types such as epithelial cells, hemocytes, and granulocytes, AMPs act as natural antibiotics, effectively killing or inhibiting the growth of a wide range of microorganisms. Upon infection or injury, marine invertebrates induce the production of AMPs, which can be secreted into the extracellular environment to protect the organism against pathogens. Molecular mechanisms through which AMPs exert their antimicrobial effects are diverse. They can disrupt the bacterial cell membrane, inhibit DNA, RNA, or protein biosynthesis, and interfere with enzyme functions. This broad-spectrum activity enables AMPs to act against bacteria, fungi, viruses, and protozoans. In response to specific pathogens, marine invertebrates activate various intracellular pathways to mount an adequate immune response. For instance, in clams and oysters, viral infections trigger RNA interference (RNAi) and an IFN-like pathway. The RNAi pathway involves the protein Dicer-2, which is known to activate innate immune pathways and cleaves viral double-stranded RNA (dsRNA) into small interfering RNAs (siRNAs). These siRNAs then bind to the RNA-induced silencing complex (RISC), which targets and binds complementary viral mRNA. Additionally, the JAK-STAT pathway is activated by cytokines called Upd, which bind to the Dome receptor, leading to the activation of JAK, STAT, and vir-1. The JAK-STAT pathway also activates PI3K and Akt, which mediate TOR signaling and induce autophagy in virus-infected cells. The Imd and Toll pathways, including proteins such as dMyD88, Tube, and Pelle, activate NF-κB effector genes, regulating cytokine levels and other transcriptional responses [218,237,238]. Upon bacterial infections, interaction between bacteria and pattern recognition receptors (PRRs) triggers the activation of multiple intracellular pathways, including NF-κB, PI3K, and Caspase, on the cell surface. This activation leads to the production of AMPs, hydrolases, proteases, as well as causes cytoskeleton remodeling, vesicle trafficking, and an increased expression of PRRs [239,240]. These intracellular responses contribute to host defense against bacterial pathogens.

## 8. Immunomodulatory Activity

Apart from their direct antimicrobial properties, AMPs from marine invertebrates possess immunomodulatory effects. They have the ability to stimulate host immune response against pathogens, thereby aiding in the defense against infections. These immunomodulatory properties make AMPs valuable therapeutic candidates for enhancing the innate immune system ability to combat microbial invasions. 

Invertebrates utilize AMPs not only to eliminate microorganisms, but also to modulate their immune response. Certain AMPs have the ability to stimulate the production of cytokines or chemokines, which attract immune cells to the site of infection [241]. Other AMPs can bind to microbial molecules or surface receptors, leading to the activation of immune cells and the generation of reactive oxygen species (ROS) or nitric oxide (NO). For instance, crustins, widely distributed among different marine crustaceans, exemplify AMPs with immune modulatory functions [106,115].

**Figure 5:**
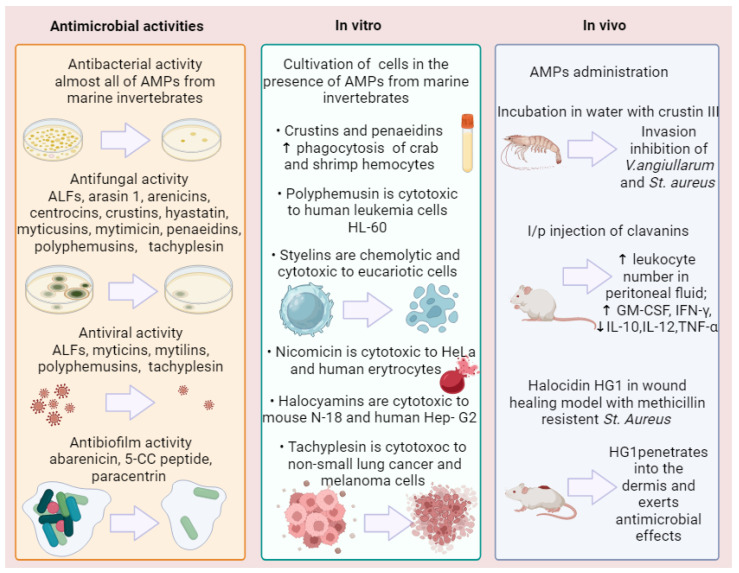
Biological activities of AMPs from marine invertebrates. An up arrow means an increase, and a down arrow means a decrease in the parameter being studied.

Several antimicrobial peptides from marine invertebrates have demonstrated the ability to inhibit the production of pro-inflammatory cytokines. Clavanin A and clavanin-MO derived from the tunicate *Styela clava* were found to possess immunomodulatory activity in a mouse model by suppressing the inflammatory response associated with sepsis, affecting immune system components and influencing cytokine modulation through the down-regulation of IL-12 and TNF-α and up-regulation of GM-CSF, IFN-γ, and MCP-1 [242]. The suppression of TNF-α production has also been demonstrated with polyphemusin. Zhang et al. have shown that the horseshoe crab polyphemusine and its analogs inhibited the binding of LPS to LPS-binding protein and thereby suppress the LPS-induced production of TNF-α by macrophages [243]. Mytilin, found in the mollusk *Mytilus edulis*, has been shown to enhance phagocytosis by transporting through hemocytes to target bacteria [214]. Myticin C has been shown to potentially influence the course of infection by inducing hemocyte chemotaxis and modulating the expression of other immune genes (Figure 5) [93].

It is important to note that the precise mechanisms by which these peptides inhibit the production of pro-inflammatory cytokines are not fully understood and may vary depending on the specific peptide and cell type involved.

Certain AMPs from marine invertebrates have also demonstrated effects on lymphocytes. Tachyplesin II, the 18-residue peptide isolated from the horseshoe crab *Tachypleus tridentatus*, inhibits T cell line-tropic (T-tropic) HIV-1 infection through its specific binding to the chemokine receptor CXCR4, which serves as a co-receptor for the entry of T-tropic HIV-1 strains. Thus, Tachyplesin II exhibits potent anti-HIV activity [244].

## 9. The Role of Marine Invertebrate AMPs in the Regulation of Interaction between Innate and Acquired Immunity Systems in Mammals

Invertebrates do not have T and B cells, clonally derived immunoglobulins, or a system of complement which form an acquired immunity [245]. However, AMPs from marine invertebrates introduced into the mammals may play a crucial role in mediating the interaction between innate and acquired immune systems. While the innate immune system provides a rapid and generalized response to pathogens, the acquired immune system offers a specific and adaptive response. AMPs, as a part of the innate immune system, act as an initial defense mechanism against invading pathogens. Beyond their direct antimicrobial properties, AMPs also possess immunomodulatory effects. They can activate immune cells, prompting the production of cytokines and chemokines that recruit and activate other immune cells at the site of infection. This immune cell activation plays a role in initiating acquired immunity. In addition, AMPs can influence the activity of cells involved in acquired immunity, such as T and B cells. Some AMPs act as chemoattractants for T cells, enhancing their activation and participation in the immune response [95]. Recombinant expression in a fish cell line of antimicrobial peptide myticin C (Myt C) from the mussel *Mytilus galloprovincialis* has conferred the protection of fish cells against two different fish viruses (enveloped and non-enveloped ones). Myt C was considered not only as an AMP, but also as the first chemokine/cytokine-like molecule identified in bivalves and one of the few examples among invertebrates [95].

AMPs interact with molecules involved in immune response regulation in mammals, such as lipopolysaccharide, lipoteichoic acid, peptidoglycan, and glucan, that are recognized by specific receptors of innate immunity and modify an immune response [127]. AMPs can bind to TLRs, modulating their activity and subsequently activating signaling pathways that lead to cytokine and chemokine production, facilitating immune cell recruitment and activation [95]. Oyster peptides in immunosuppressed mice by cyclophosphamide restored the indexes of the thymus, spleen, and liver, stimulated cytokines secretion, and promoted the relative mRNA levels of Th1/Th2 cytokines (IL-2, IFN-γ, IL-4, and IL-10) and the NF-κB signaling pathway [246]. 

Furthermore, AMPs can serve as adjuvants, enhancing vaccine efficacy. Adjuvants stimulate the immune system, boosting the immune response to vaccines. Some AMPs have demonstrated the ability to enhance antibody production in response to vaccines targeting bacterial pathogens [247]. Marine invertebrates are a great source of molecules with a wide range of activities, including adjuvanticity [23].

Despite the lack of adaptive immunity in marine invertebrates, there is a growing evidence of their immune memory based on cellular and humoral factors. In particular, an increased expression of antilipopolysaccharide factors after primary stimulation has been found, which provides a long-term humoral protection in the crab *Eriocheir sinensis* [248].

In addition, the role of arenicin from the marine polychaete *Arenicola marina* in the modulation of the complement system has been shown [81,82,249,250].

The role of AMPs in regulating the interplay between innate and acquired immunity in mammals is complex and diverse. They strengthen the innate immune response and modulate the activity of cells involved in acquired immunity. Understanding the underlying mechanisms of AMPs’ immunomodulatory effects in marine invertebrates holds significant potential for the development of novel therapies and vaccines against infectious diseases [247,251].

Marine invertebrates are considered as promising sources of bioactive molecules and drugs, and AMPs are considered as deserving more attention [24,25].

## 10. Alternative Functional Properties of AMPs Derived from Marine Invertebrates

In addition to their well-known antimicrobial properties, AMPs in marine invertebrates exhibit a diverse range of alternative functional properties, thereby expanding their potential roles beyond antimicrobial defense. One such alternative function of AMPs is their capacity to act as signaling molecules. For instance, tachyplesin have been demonstrated to regulate cell proliferation, differentiation, and apoptosis [139]. In some cases, these signaling effects may be linked to their antimicrobial properties, such as the ability of hydralysins from Cnidaria to disrupt bacterial membranes [252]. However, in other cases, these signaling effects appear to be independent of HPD antimicrobial activity.

AMPs also possess the ability to bind to bacteria. It has been shown that the crustin SpCrus5 from the mud crab *Scylla paramamosain*, containing a typical cysteine-rich domain at the N-terminus, a conserved WAP domain in the center, and a special GRR at the C-terminus, participate in antibacterial immune responses [114,115]. Crustins have been shown to enhance phagocytosis through bacteria opsonization, thereby providing protection in vivo [127].

Furthermore, AMPs derived from marine invertebrates have been found to possess wound healing properties. These AMPs promote cell migration, proliferation, angiogenesis, and tissue regeneration. For instance, myticin C, a cationic peptide, found in the marine mussel *Mytilus galloprovinciali*, has been shown to accelerate re-epithelialization and angiogenesis, thereby promoting skin wound healing in mice [90]. Halocidin from the tunicate *Halocynthia aurantium* has been investigated as a potential topical antibiotic in a mouse model of surgical wound infected with methicillin-resistant *S. aureus*. Results indicated that topically administered halocidin successfully penetrated the dermis at the infection site, exerting its antimicrobial effects [253].

As already mentioned above, AMPs also exhibit immunomodulatory properties. They are capable of modulating the activity of immune cells in mammals, including macrophages, dendritic cells, and T cells. Some AMPs, for example, clavanines and polyphemusines, have been shown to down-regulate the production of cytokines, such as IL-12 and TNF-α, which promote inflammation and an increase in IFN-γ, enhancing antiviral immunity [239,240]. This ability of clavanines to reduce inflammation and induce antiviral protection may be very important in the treatment of viral diseases. Additionally, the ability of AMPs to activate or inhibit the complement system in vitro has been demonstrated; moreover, these effects have been shown to be concentration-dependent [81,82].

It is noteworthy that marine invertebrates produce AMPs not only for their own protection, but also for safeguarding symbiotic bacteria, which can constitute a significant proportion of their mass, reaching up to 37%. Symbiotic bacteria are believed to produce substances with a cytotoxic activity to defend against predators such as fish or sea urchins and prevent colonization by macroorganisms [254].

Another alternative function of AMPs in marine invertebrates is their potential as anticancer agents. Some AMPs have displayed a selective cytotoxicity against cancer cells while sparing normal cells, making them potential candidates for cancer therapeutics. Polyphemusin III has exhibited cytotoxic effects on human leukemia cells (HL-60) by permeabilizing the cell membrane [133]. Others are cytotoxic for cancer and normal cells. For example, nicomicins from the marine polychaeta *Nicomache minor* possessed cytotoxicity against cancer cells (HeLa) and normal adherent cells as well as toward human erythrocyte [58]. Tachyplesins have been found to be toxic toward human non-small-cell lung cancer cells and normal cells [137,138,139]. Backbone cyclized analogs of tachyplesin 1 had increased selectivity toward melanoma cells and its analog has shown an ability to enter cells with a high efficacy and a low toxicity [255]. 

In summary, the alternative functional properties of AMPs in marine invertebrates extend beyond their antimicrobial defense role and encompass signaling, immunomodulation, wound healing, and potential anticancer applications. Advancing our understanding of mechanisms underlying these alternative effects of AMPs holds promise for the development of novel therapies for treatments of various diseases.

## 11. Prospects for the Therapeutic Use of Marine Invertebrate AMPs

AMPs derived from marine invertebrates offer a significant potential for therapeutic applications due to their broad-spectrum antimicrobial activities, a low toxicity, and versatile functional properties. The following are some potential prospects and applications for the therapeutic use of AMPs:

### 11.1. Anti-Infective Agents

AMPs have demonstrated a promising efficacy against various pathogenic microorganisms, including bacteria, viruses, fungi, and protozoans. Their broad-spectrum activities and a low propensity for resistance development make them attractive candidates for novel antimicrobial therapeutics [68,256]. By modifying naturally occurring AMPs, such as holothuroidin 2 from the Mediterranean sea cucumber, more potent synthetic peptides, exemplified by their enhanced activity against *Listeria monocytogenes*, have been created [100]. Effective antibiotics against *Listeria monocytogenes*, which has a high mortality rate and forms biofilms on diverse surfaces, would have a significant clinical impact [257,258]. The complex mechanism of action mediated by various molecular targets coupled with the ability to rapidly destroy pathogen cells prevent the formation of effective mechanisms for the development of resistance to AMPs. These compounds may be useful in the treatment of chronic infections because they are able to destroy persistent cells and penetrate the biofilm matrix [259]. Although microorganisms are able to develop resistance to AMPs by decreasing transmembrane potential, through the covalent modification of membrane lipids, LPS, LTAs, and through the secretion of proteases, etc. [260], the removal of the peptides from the medium usually leads to the rapid restoration of their sensitivity, which may indicate a high fitness cost of such a resistance [261].

### 11.2. Antibiofilm Activity

A wide range of AMPs from marine invertebrates and their analogs have an antibiofilm activity against Gram-positive and Gram-negative bacteria, in particular, abarenicin from *Abarenicola pacifica* and its analog Ap9, the 5-CC peptide from the sea urchin *Paracentrotus lividus,* and its analog paracentrin 1 [51,100,101,102]. Their ability to prevent biofilm formation, including biofilms formed by drug-resistant strains, can increase the effectiveness of interventions in surgery, dentistry, and endoprosthetics. Effective antibiotics against *Listeria monocytogenes*, which has a high mortality rate and forms biofilms on diverse surfaces, would have a significant clinical impact [257,258].

### 11.3. Wound Healing Agents

AMPs have shown the ability to expedite wound healing in animal models, potentially by stimulating angiogenesis and tissue regeneration. The development of AMP-based therapies could hold great promise in the treatment of chronic wounds and tissue injuries [92].

### 11.4. Immunomodulatory Agents

AMPs possess immunomodulatory properties, including the activation of immune cells and the promotion of cytokine production. These characteristics indicate that AMPs could serve as adjuvants for vaccines or immune-modulating therapies for patients diagnosed with cancer, autoimmune diseases, and allergies.

### 11.5. Anticancer Agents

Some AMPs exhibit a selective cytotoxicity toward cancer cells while sparing healthy cells. This suggests that AMPs may serve as novel anticancer therapeutics, either alone or in combination with existing drugs. For instance, halocyamines, tetrapeptides derived from the morula cells of the solitary ascidian *Halocynthia roretzi*, have displayed a pronounced activity against mouse neuroblastoma N-18 cells and human hepatoma Hep-G2 cells [262].

### 11.6. Food Preservatives

Certain AMPs have demonstrated an antimicrobial activity against foodborne pathogens and spoilage organisms. Using AMPs as food preservatives, it is possible to prolong the shelf life of food products and mitigate the risk of foodborne illnesses.

### 11.7. Agricultural Applications

AMPs hold promise for potential applications in agriculture as biopesticides, as they can selectively target pathogens while displaying low toxicity toward non-target organisms. Additionally, the evaluation of AMP structures enables the possibility of the development of transgenic aquacultures of economically significant marine invertebrates, such as mussels and crustaceans. Given the decline in these species due to emerging diseases, largely influenced by climate change, this knowledge could aid in maintaining biodiversity and economic stability for human well-being. 

Despite many advantages and potential applications, the commercialization of marine invertebrate AMPs as next-generation antibiotics faces several challenges: a relatively high toxicity, a low resistance to proteolytic degradation, binding to plasma proteins, a low bioavailability, and a high cost of chemically synthesized and recombinant drugs [220,263,264,265,266]. The arguments pro- and contra-AMPs as therapeutic agents are summarized in Table 2.

To date, most of the above points are under development. A considerable decrease in the cost of AMP production will stimulate scientists to move from fundamental studies to preclinical and clinical trials. Undoubtedly, the first anti-infective marine AMP-based agent will be introduced into the world’s medical practice over the next ten years.

## 12. Conclusions

Antimicrobial activities of marine invertebrate AMPs encompass a variety of mechanisms, highlighting the evolutionary adaptations of these organisms in countering diverse microbial threats. The complexity of these mechanisms underscores the potential therapeutic value of AMPs from marine invertebrates across a broad spectrum of applications, including anti-infective agents, wound healing, and anticancer therapy. 

Mechanisms of antimicrobial activities exhibited by AMPs in marine invertebrates are diverse and intricate, representing remarkable evolutionary adaptations of these animals to a wide range of microbial threats. The multifaceted nature of marine invertebrate AMPs enables them to combat pathogens through various mechanisms, including membrane disruption, cell penetration, and interference with intracellular molecular targets. Moreover, the alternative functional properties of AMPs, such as their immunomodulatory effects and wound healing capabilities, further enhance their therapeutic potential.

The therapeutic use of AMPs from marine invertebrates holds great promise to address pressing healthcare challenges. As antimicrobial resistance continues to rise, a broad-spectrum activity and a low propensity for resistance development exhibited by AMPs make them attractive candidates for the development of novel anti-infective agents. Furthermore, their ability to promote wound healing and tissue regeneration suggests their potential application in chronic wound management and regenerative medicine.

In the field of anticancer therapy, a selective cytotoxicity of certain AMPs against cancer cells offers a promising avenue for the development of targeted anticancer agents. Combining the unique properties of AMPs with existing treatment modalities may lead to the development of more effective and personalized anticancer therapies.

While the therapeutic potential of AMPs from marine invertebrates is evident, further research is essential to unlock their full capabilities. Molecular insights into mechanisms of their action, optimizing their efficacy, and ensuring their safety profile are crucial steps in harnessing the therapeutic potential of these molecules. Additionally, exploring the vast biodiversity of marine invertebrates and their associated microbiomes can unveil novel AMPs with unique properties and therapeutic applications.

Leveraging their remarkable antimicrobial and alternative functional properties will allow us to address significant global health challenges. Continued research and development efforts in this area hold tremendous promise for the future of medicine, where AMPs may play a pivotal role in combating infections, promoting wound healing, and advancing personalized therapies. However, further research is essential to gain a comprehensive understanding of underlying mechanisms of action and to maximize the therapeutic potential of AMPs in these contexts.

## Figures and Tables

**Table 1:** Brief description and structural characterization of AMPs from marine invertebrates.

Cys-Containing Peptides Stabilized by Disulfide Bonds						
No.	AMP/Family	Origin	Structure *	Activities **	Additional Data	References
1	Anti-LPS factors (ALFs)	Hemocytes of the *Tachypleus tridentatus* and *Limulus polyphemus* horseshoe crabs	114–124 a.a., 2Cys, αββββαα	G+, G−, F, V	Several subfamilies with different pI and activity spectra; only cationic ALFs exhibit antimicrobial activity	[26]
2	Aurelin	Mesoglea of the *Aurelia aurita* jellyfish	40 a.a., 6Cys, αα	G+, G−	Homologous to the K+ channel blockers from sea anemones (BgK, ShK)	[27,28]
3	Big defensins	Hemocytes of the *Tac-hypleus tridentatus* king crab, mussels, and the *Branchiostoma japonicum* lancelet	79–94 a.a., two domains; the first one is hydro- phobic βααβ, the second one contains 6Cys, βββαβ	G+, G−, F	The spatial structure of the *C*-terminal domain is similar to that of β-defensins of vertebrates	[29,30]
4	Macins: hydramacin, neuromacin, mytimacin, thero- macin, etc.	Entoderm of cnidarians and mesothelium and nervous tissue of mollusks and oligochaetes	54–78 a.a., 6/8/12Cys, βααββ (knottin)	G+, G−	Neuromacin participates in regeneration of nervous tissue of leeches	[31]
5	Myticusins	Hemocytes of the *Mytilus coruscus* mussel	104 a.a., 10Cys	G+, (G−, F)	No homologs among known AMPs	[32,33]
6	Mytilins	Hemolymph of the *Mytilus edulis* mussel	32–34 a.a., 8Cys, αββ	G+, V, (G−)	The spatial structure is similar to that of Sαβ-defensins	[34,35]
7	Mytimycin	Hemocytes of the *Mytilus edulis* mussel	54 a.a., 12Cys	F	No homologs among known AMPs	[36]
8	Myticins	Hemocytes of the *Mytilus galloprovincialis* mussel	40 a.a., 8Cys	G+, V, (G−, F)	The spatial structure has not yet been solved	[8]
9	Tachystatins	Hemocytes of the *Tachypleus tridentatus* horseshoe crab	41–44 a.a., 6Cys, βββ	G+, G−, F, (H)	Primary and spatial structures and chitin-binding properties are similar to those of the spider agatoxins	[37,38]
10	Tachycitin	Hemocytes of the *Tachypleus tridentatus* horseshoe crab	73 a.a., 10Cys, *C*-terminal amidation; two domains: βββ, ββα	G+, G−, F	The *C*-terminal domain is homologous to chitin-binding domains of chitinases; amidation is crucial for antibacterial activity	[39]
**β-Hairpin Peptides**						
**No.**	**AMP/Family**	**Origin**	Structure *	Activities **	**Additional Data**	**References**
11	Arenicin-1 and arenicin-2	Coelomocytes of the *Arenicola marina* polychaete	21 a.a., 2Cys, ββ	G+, G−, F, H	Dimerization of arenicin plays a key role in the cytotoxicity but not in the antibacterial activity	[40,41,42,43]
12	Tachyplesins and polyphemusins	Hemocytes of the *Tachypleus tridentatus* and *Limulus Polyphemus* horseshoe crabs	17–18 a.a., 4Cys, ββ	G+, G−, F, V, P, T, H	Affinity to LPS and chitin, strong hemolytic effect	[44,45,46,47]
13	Capitellacin	The marine polychaeta *Capitella teleta*	20 a.a., 4Cys, ββ	G+, G−	A high homology with tachyplesins and polyphemusins	[48,49]
14	Alvinellacin	Coelomocytes of the extremophile marine polychaeta *Alvinella pompejana*	22 a.a., 4Cys, ββ	G+, G−	The first AMP from a deep-sea organism	[50]
15	Abarenicin	The marine polychaeta *Abarenicola pacifica*	21 a.a., 4Cys, ββ	G-	A high antibiofilm activity	[51]
16	UuBRI-21	The marine polychaeta *Urechis unicinctus*	21 a.a., 4Cys, ββ	G-	-	[51]
**Linear α-Helical Peptides**						
**No.**	**AMP/Family**	**Origin**	Structure *	Activities **	**Additional Data**	**References**
17	Halocydin	Hemocytes of the *Halocynthia aurantium* sea squirts	18 a.a. + 15 a.a. (disulfide bond), α+α	G+, G−	Covalent heterodimer. The Trp-Leu-Asn *N*-terminal tripeptide of the long chain (18 a.a.) plays a crucial role in maintaining antimicrobial activity	[52]
18	Hedistin	Coelomocytes of the marine polychaeta *Nereis diversicolor*	22 a.a., α+α (helix-turn- helix motif)	G+, G−	Contains 2 bromotryptophan residues and *C*-terminal amidation	[53,54]
19	Dicynthaurin	Hemocytes of the *Halo- cynthia aurantium* sea squirts	30 a.a. + 30 a.a. (disulfide bond), α+α	G+, G−	Covalent homodimer; the activity of the monomer is equal to that of the full-size molecule	[55]
20	Clavanins and clavaspirin	Hemocytes of the *Styela clava* sea squirts	23 a.a., His-rich and Phe-rich (clavaspirin is enriched with His), *C*-terminal amidation	G+, G−, F, (H)	The precursor protein is similar to prepropeptides of several amphibian AMPs; pH-dependent mechanism of action; clavaspirin is characterized by significant hemolytic activity; Phe residues have no effect on the antimicrobial properties	[56]
21	Styelins	Hemocytes of the *Styela clava* ascidian	31–32 a.a., *C*- amidated, Pherich, 6-bromotrypto-phan, dihydroxy-arginine, dihydroxylysine, dihydroxy-phenylalanine	G+, G−, H	Homologous to cecropins of insects and pleurocidin of the *Pseudopleuronectes americanus* winter flounder; activity is maintained at high ionic strength	[56,57]
22	Nicomicins	The marine polychaeta *Nicomache minor*	33 a.a., combining an amphipathic *N*-terminal α-helix and *C*-terminal extended part with a six-residue loop stabilized by a disulfide bridge	G+, T	Share similarities in both primary and secondary structure with amphibian AMPs	[58]
23	Centrocins	Coelomocytes of the *Strongylocentrotus droebachiensis* green sea urchin	12 a.a. + 30 a.a., (disulfide bond), bromotryptophan	G+, G−, F	Covalent heterodimers	[59]
24	Polaricin	*Amphitritides* sp.	19 a.a.; one cysteine (Cys10) engaged in one intermolecular disulfide bridge (Cys10-Cys10)	*V. alginolyticus*	Forms the non-covalent homodimer	[60]
**Linear Peptides Enriched in Particular Amino Acid Residues**						
**No.**	**AMP/Family**	**Origin**	Structure *	Activities **	**Additional Data**	**References**
25	Antibacterial 6.5-kDa protein	Hemocytes of the *Carci- nus maenas* green crab	6.5 kDa, Gly-rich	G+, G−	–	[61]
26	Astacidin-2	Hemocytes of the *Pacifastacus leniusculus* signal crayfish	14 a.a., Gly-rich	G+, G−	–	[62]
**Mixed-Type Peptides Containing Domains of Different Structures**						
**No.**	**AMP/Family**	**Origin**	Structure *	Activities **	**Additional Data**	**References**
27	Arasin 1	Hemocytes of the *Hyas araneus* great spider crab	37 a.a., two domains: Pro-rich and *C*-terminal 4Cys/Pro-rich	G+, G−, F	Deletion of the Cys-containing *C*-terminal domain does not affect the antimicrobial activity	[63]
28	Hyastatin	Hemocytes of the *Hyas araneus* great spider crab	114 a.a., *C*-terminal amidation; three domains: the first is Gly-rich, the second is Pro/Arg-rich, and the third contains 6 Cys	G+, G−, F	–	[64]
29	Callinectin	Hemocytes of the *Callinectes sapidus* blue crab	32 a.a., 4Cys, Pro-rich, *C*-terminal amidation, 3 variants of Trp oxidative modifications	G−	Homologous to arasin-1	[65]
30	Crustins (6–22 kDa)	Crustaceans (penaeid shrimp, freshwater prawns, lobsters, crayfishes, crabs, etc.)	56–201 a.a., 1–3 domains: conservative *C*-terminal 12Cys WAP-domain (+optional WAP, Gly-, Cys-, Pro/Arg-, and AAA-rich)	G+, G−, F	Several subfamilies with different domain organizations; inhibitors of protease activities	[66]
31	Penaeidins	Hemocytes of penaeid shrimps	47–67 a.a., pGlu, *C*-terminal amidation; two domains: the first is Pro-rich and the second contains 6Cys, α-helix	G+, F, (G−)	The *C*-terminal domain is homologous to plant chitin-binding proteins; the absence of the *C*-terminal amidation impairs antibacterial activity	[67]

* The following elements of the primary and secondary structures are indicated: the number of amino acid residues in a chain (a.a.), the number of cysteine residues (nCys), the number and positions of α-helixes and β-sheets, the presence of the *C*-terminal amide (*C*-amide), *N*-terminal pyroglutamate (pGly), glycosylated residues of serine and threonine (*O*-glyc), and clusters of aromatic amino acid residues (AAA). Designations of the structural elements, which occur in only a part of the representatives of a family, are given in parentheses. ** The data on the activities toward the following targets are given: Gram-positive bacteria (G+), Gram-negative bacteria (G−), fungi (F), protozoa (P), viruses (V), tumor cells (T), and mammalian erythrocytes (H). Low or uncharacteristic activities for most of the representatives of the concrete family toward the indicated group of targets are given in parentheses.

**Table 2:** Marine invertebrate AMPs as therapeutic agents: the pros and cons.

Strengths	Weaknesses
Effective against microorganisms resistant to conventional antibiotics	Low proteolytic stability
Broad activity range	Activity against host cell membranes
No toxic metabolism by-products	Still prone to resistance development
High fitness cost of acquired resistance	Impaired pharmacokinetics
No cross-resistance with conventional antibiotics	High production cost

## Data Availability

Not applicable.
